# Combination of Ipilimumab and Adoptive Cell Therapy with Tumor-Infiltrating Lymphocytes for Patients with Metastatic Melanoma

**DOI:** 10.3389/fonc.2018.00044

**Published:** 2018-03-02

**Authors:** John E. Mullinax, MacLean Hall, Sangeetha Prabhakaran, Jeffrey Weber, Nikhil Khushalani, Zeynep Eroglu, Andrew S. Brohl, Joseph Markowitz, Erica Royster, Allison Richards, Valerie Stark, Jonathan S. Zager, Linda Kelley, Cheryl Cox, Vernon K. Sondak, James J. Mulé, Shari Pilon-Thomas, Amod A. Sarnaik

**Affiliations:** ^1^Sarcoma Department, Moffitt Cancer Center, Tampa, FL, United States; ^2^Immunology Department, Moffitt Cancer Center, Tampa, FL, United States; ^3^Department of Surgery, University of New Mexico, Albuquerque, NM, United States; ^4^NYU Langone Medical Center, New York, NY, United States; ^5^Department of Cutaneous Oncology, Moffitt Cancer Center, Tampa, FL, United States

**Keywords:** adoptive cell therapy, melanoma, ipilimumab, checkpoint inhibitor, immunotherapy, tumor-infiltrating lymphocytes

## Abstract

**Purpose:**

Adoptive cell therapy (ACT) using tumor-infiltrating lymphocytes (TIL) for metastatic melanoma can be highly effective, but attrition due to progression before TIL administration (32% in prior institutional experience) remains a limitation. We hypothesized that combining ACT with cytotoxic T lymphocyte-associated antigen 4 blockade would decrease attrition and allow more patients to receive TIL.

**Experimental design:**

Thirteen patients with metastatic melanoma were enrolled. Patients received four doses of ipilimumab (3 mg/kg) beginning 2 weeks prior to tumor resection for TIL generation, then 1 week after resection, and 2 and 5 weeks after preconditioning chemotherapy and TIL infusion followed by interleukin-2. The primary endpoint was safety and feasibility. Secondary endpoints included of clinical response at 12 weeks and at 1 year after TIL transfer, progression free survival (PFS), and overall survival (OS).

**Results:**

All patients received at least two doses of ipilimumab, and 12 of the 13 (92%) received TIL. A median of 6.5 × 10^10^ (2.3 × 10^10^ to 1.0 × 10^11^) TIL were infused. At 12 weeks following infusion, there were five patients who experienced objective response (38.5%), four of whom continued in objective response at 1 year and one of which became a complete response at 52 months. Median progression-free survival was 7.3 months (95% CI 6.1–29.9 months). Grade ≥ 3 immune-related adverse events included hypothyroidism (3), hepatitis (2), uveitis (1), and colitis (1).

**Conclusion:**

Ipilimumab plus ACT for metastatic melanoma is feasible, well tolerated, and associated with a low rate of attrition due to progression during cell expansion. This combination approach serves as a model for future efforts to improve the efficacy of ACT.

## Introduction

The treatment of metastatic melanoma has evolved greatly over the past decade. The standard treatment in these patients now includes agents that enhance the immune response to melanoma. Immunotherapy using high-dose interleukin-2 (IL-2) resulted in a 17% objective response rate, with 8% demonstrating a complete response (1), but has never been shown to improve overall survival. Rosenberg et al. developed a strategy to culture, expand, and reinfuse tumor-infiltrating lymphocytes (TIL) combined with IL-2 for patients with metastatic melanoma after a preparative lymphodepleting regimen of cyclophosphamide and fludarabine (2–5). The objective response rate for this adoptive cell therapy (ACT) at the National Cancer Institute (NCI) was 55% for those that received TIL, with up to 22% demonstrating a complete response (4).

Due to the intensive laboratory and clinical requirements, ACT has been conducted at only a few centers outside the NCI. Investigators at MD Anderson Cancer Center and Sheba Medical Center (Tel Aviv, Israel) reported objective response rates of 48% and 40%, respectively, for melanoma patients who received TIL (6, 7). Initial experience at our institution with ACT for metastatic melanoma involved a trial of 19 patients. Progression of disease occurred in six patients (21%) before receiving TIL, leaving 13 patients who received the TIL infusion and IL-2. The response rate for those treated was 38%, but it was only 26% by intention to treat analysis including all patients who underwent resection of tumor for TIL harvest (8). The only other center reporting data by intention to treat (Sheba Medical Center) reported a similar 29% response rate. At both centers, dropout due to progression during the time required for TIL production (generally 6 weeks) resulted in a significant percentage of patients—nearly one-third—not receiving the full treatment.

Systemic immunotherapy using antibodies that block immune checkpoints to boost a tumor-specific immune response has been shown to improve overall survival for patients with unresectable metastatic melanoma and is now a standard treatment for most such patients (9, 10). The first checkpoint inhibitor blocking therapy approved was ipilimumab, an antibody that inhibits cytotoxic T lymphocyte-associated antigen 4 (CTLA-4) (9, 11–15). When used as monotherapy, an overall response rate of up to 11% was seen for patients with metastatic disease (9, 16). Ipilimumab has been combined with a monoclonal antibody against the programmed death-1 (PD-1) receptor, which resulted in a 58% objective response rate and a 58% 3-year overall survival rate (10). Severe or life-threatening immune-related adverse events occur in up to 54% of patients treated with combination therapy, compared with 15–24% with ipilimumab alone (9, 14, 16, 17).

In this report, we describe the results of a trial designed to assess the safety and feasibility of the addition ipilimumab to ACT for metastatic melanoma. The rationale for this approach is threefold. First, combining checkpoint inhibition with ACT could decrease dropout associated with disease progression since patients receive an active therapy during the TIL production phase. Second, we hypothesized that the efficacy of ACT would be enhanced by incorporating an immunomodulator proven to improve survival. Finally, the use of a checkpoint inhibitor before the resection to harvest TIL could result in more efficacious TIL needing reduced time in culture to yield the same degree of *ex vivo* expansion, with enhanced antitumor reactivity (18, 19). The purpose of this trial was to test the hypothesis that combining checkpoint inhibition with ipilimumab was safe and feasible, as well as to obtain a preliminary evaluation of its impact on TIL efficacy.

## Methods

### Patient and Treatments

Eligible patients had unresectable stage-III or -IV melanoma with at least one lesion amenable to TIL harvest, age greater than 18 years, ECOG status 0–1, adequate organ function, and no prior treatment with ipilimumab (prior ACT was permitted). Patients were excluded if they had active systemic infections, serologic evidence of HIV, Hepatitis B or C virus, HTLV I or II, or syphilis. Pregnant or breast-feeding women were excluded. Sexually active patients were required to use two forms of effective contraception from the initiation of ipilimumab until 6 months after ACT. All eligible patients signed an IRB-approved informed consent (clinical protocol NCT# 01701674).

**Figure 1:**
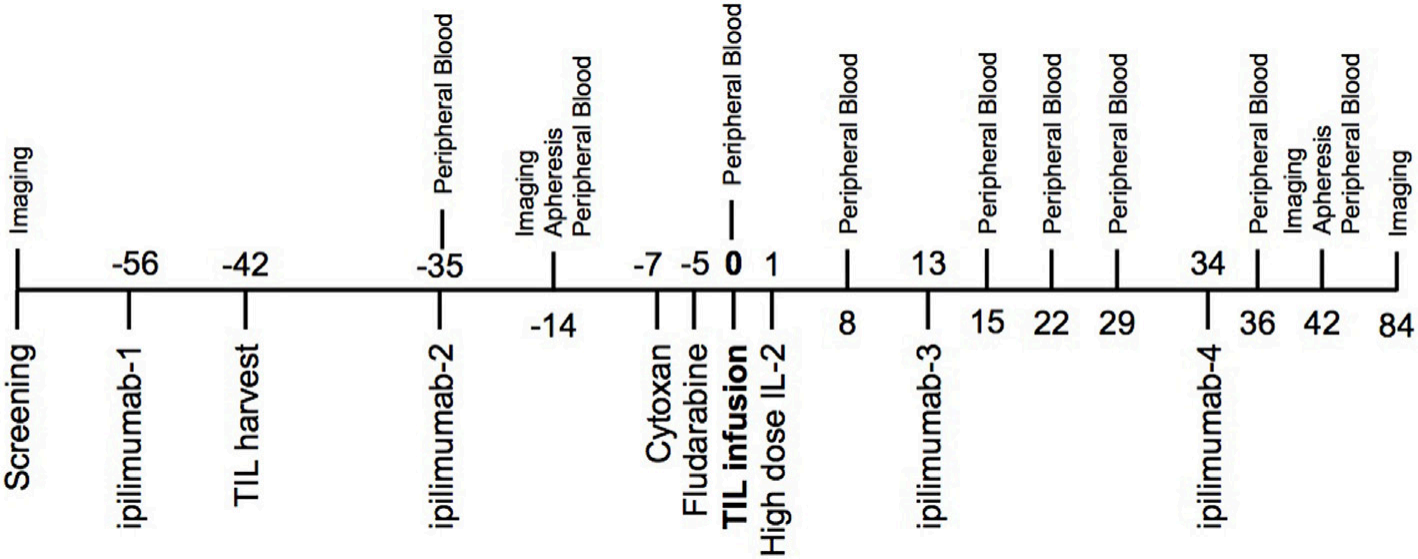
Trial schema—two doses of ipilimumab were given prior to tumor-infiltrating lymphocyte (TIL) infusion and two doses were given after cell infusion. One of the pre-infusion doses was given 2 weeks prior to the TIL harvest. Imaging time points included computed tomography chest, abdomen, and pelvis with MRI brain. Peripheral blood drawn at indicated time points was used for correlative studies.

The treatment schema (Figure 1) began with the first dose of ipilimumab (3 mg/kg) administered 2 weeks prior to harvest of tumor at least 1 cm^3^ to serve as the source of TIL. A second dose of ipilimumab was administered 1 week following tumor harvest. All subjects underwent restaging at day −14 relative to the planned TIL infusion. Treatment was discontinued if patients had new brain metastases or significantly decreased performance status due to tumor progression. Otherwise, cyclophosphamide (60 mg/kg/day) and mesna (20 mg/kg) were given intravenously on day −7 and day −6 relative to the anticipated TIL infusion date. Fludarabine (25 mg/m^2^) was given daily intravenously from day −5 to day −1. The TIL infusion was administered on day 0 and high-dose IL-2 (aldesleukin, Prometheus Laboratories Inc., San Diego, CA, USA) was started at 720,000-IU/kg IV bolus every 8–16 h for up to 15 doses, beginning 12–16 h after TIL infusion. The third dose of ipilimumab was given after recovery from high-dose IL-2, approximately day +13. The final dose of ipilimumab was given 3 weeks later. Apheresis was performed on days −14 and +42 via a two-armed peripheral approach or using a temporary central venous catheter.

During the preparative chemotherapy regimen and high-dose IL-2 therapy, patients underwent routine vital sign monitoring and laboratory evaluation to include complete blood count (CBC), complete metabolic panel (CMP), and lactate dehydrogenase (LDH) level. Computed tomography (CT) scans of the chest, abdomen, and pelvis on days +42 and +84 were assessed for clinical response by RECIST v1.1 criteria using the baseline (pre-IPI) measurements as a reference. Subsequent response evaluations occurred at 3-month intervals for 2 years, followed by 6-month intervals for 2 years and annually thereafter.

### Preparation and Evaluation of TIL for Infusion

Following tumor resection, tissue was transported in a sterile manner to the laboratory where tumor fragments were minced into 1-mm cubes. tumor fragments were placed individually into at least 12 wells of a 24-well plate in culture media containing 6,000 IU/mL of IL-2. Fragments were monitored for growth every 2–3 days for up to 5 weeks. TIL were expanded to a new well of a 24-well plate when they became 80% confluent, keeping TIL derived from each fragment separate. Media containing 6,000-IU/mL IL-2 was replenished every 3–4 days in order to maintain the proliferation of the TIL.

When available, a small amount of excess tumor not used to grow TIL was digested into a single-cell suspension and cyropreserved to generate target cells for ELISA assays using the following method. The tumor was first minced in similar fashion as above, then gently stirred in digestion media containing collagenase (type II and type IV), hyaluronidase, and DNAase (Fisher Scientific Co., Pittsburgh, PA, USA). After digestion, the cell suspension was filtered to remove undigested tumor and connective tissue to generate a single-cell suspension. Intact cells were enriched with a Ficoll-Hypaque (GE Healthcare Bio-Sciences, Pittsburgh, PA, USA) gradient, and viable tumor cells were enumerated by trypan blue exclusion.

Tumor-infiltrating lymphocytes from the fastest-growing fragments were assessed for antitumor reactivity by overnight coculturing with autologous (when available) and/or HLA-matched and HLA-mismatched tumor cells in a 1:1 ratio. Interferon-gamma (IFN-γ) in culture supernatants was measured by ELISA. TIL were determined to be reactive if in a comparative assay autologous tumor coculture yielded at least 200 pg/mL of IFN-γ and was at least twofold higher compared with HLA-mismatched tumor coculture. When autologous tumor digest was not available, HLA-matched tumor lines were substituted for the coculture.

Tumor-infiltrating lymphocyte pools containing 3–6 × 10^7^ of the highest IFN-γ-producing TIL were selected and pooled for the rapid expansion protocol (REP). TILs were cultured in up to 60 T-175 flasks (1 × 10^6^ cells per flask) at a 1:200 ratio with irradiated allogeneic feeder cells. IL-2 at a concentration of 3,000 IU/mL and OKT3 (Ortho Biotech) at a concentration of 30 ng/mL were added to the flasks. After 7 days, flasks were pooled into 3-L culture bags (American Fluoroseal, Gaithersburg, MD, USA) so that a minimum TIL concentration of 3 × 10^5 ^/mL was added to each bag. Bags were monitored for the next 7 days and split as needed to maintain the TIL concentration at 2 × 10^6 ^/mL. The cells were harvested, washed, and concentrated to less than 1.5 L. Viability of the expanded product was performed using acridine orange (AO) and propidium iodide (PI) dyes. Live cells were enumerated using a Cellometer Auto 2000 (Nexcelom Bioscience, Lawrence, MA, USA). The final product was tested for sterility and then gravity dripped into the patient at a rate of 300 mL per hour. For flow cytometry, cells from the final product were stained with 7-AAD, CD3-FITC, CD4-PE, CD45-V500, and CD8-APC. Data were acquired on a FACSCanto (BD Biosciences, San Jose, CA, USA) and analyzed using FlowJo Software (Treestar, Ashland, OR, USA).

### TCRβ Receptor Diversity Analysis by Deep Sequencing

An apheresis sample was collected at 6 weeks after TIL infusion. Post-REP TIL product and apheresis specimens were subjected to TCR clonotyping (ImmunoSEQ™, Adaptive Biotechnologies) to track persistent T-cell clones *in vivo*. DNA extraction of post-REP TIL product and leukopheresis product at 6 weeks after TIL infusion was performed following the DNeasy Blood & Tissue Kit (Qiagen, Valencia, CA, USA). Samples were analyzed by high-throughput sequencing of the TCRβ CDR3 region using the ImmunoSEQ immune profiling system at the deep level (Adaptive Biotechnologies, Seattle, WA, USA). Bioinformatic analysis was performed on the sequencing data using the ImmunoSEQ platform and included a determination of the number and sequence of each of the productive unique Vβ and Jβ genes identified within each sample and the degree of clone sharing between samples.

### Statistical Analysis

For comparison of the final infusion product and the final infusion phenotype, an unpaired *t*-test with Welch’s correction was performed. A Mann–Whitney *U* test was performed for the comparison between the repertoire overlap in responders and non-responders. Differences in age, gender, stage, or prior treatment were not investigated using a multivariate analysis given the number of patients in this trial. All statistical computations were performed using GraphPad Prism v7 (GraphPad Software, La Jolla, CA, USA).

## Results

Thirteen patients were enrolled from January 2013 through December 2015 (Table 1). All patients had stage-IV melanoma (1 M1a, 2 M1b, and 10 M1c). There were seven male patients (53.8%) and the median age was 51 years (range 22–70 years). Most patients (76.9%) had no prior therapy for metastatic disease. Two patients had prior targeted therapy (combined BRAF/MEK inhibition). There was one patient with ocular melanoma, and this patient had prior treatments including radioembolization, anti-PD-L1, and ACT.

**Table 1:** Patient demographics.

Patient no.	Age	Sex	ECOG performance status	M stage	LDH level(>ULN)	Prior treatment for metastatic disease	Number of IL-2 doses	Response at 12 weeks	Response at 1 year
1	61	F	1	M1C	323	None	6	PR	PR^a^
2	46	M	1	M1C	444	BRAF/MEK	8	PR	PR
3	70	F	1	M1B	196	None	5	PR	PR
4	51	M	0	M1C	353	None	4	PR	PR
5	68	M	1	M1C	258	None	2	PR	PD
6	57	F	1	M1C	468	None	5	SD	PD
7	66	M	1	M1C	188	None	3	SD	PD
8	46	F	1	M1C	166	BRAF/MEK	5	SD	PD
9	37	M	1	M1C	492	None	7	PD	PD
10	26	F	0	M1C	1,887	ACT, anti-PD-L1, Chemotherapy	2	PD	PD
11	39	M	1	M1A	199	None	4	PD	PD
12	22	M	0	M1C	209	None	6	PD	PD
13	55	F	1	M1B	207	None	n/a	Not treated	Not treated

*Patient number is not sequential by enrollment*.

*^a^Complete response (CR) at 52 months*.

### Safety and Feasibility

The primary endpoint of feasibility (prespecified as ≥60% of patients receiving TIL infusion and ≥2 doses of ipilimumab) was met, with 92.3% (12 of 13) patients qualifying. All patients received at least two doses of ipilimumab. The single patient who did not receive a TIL infusion progressed within 6 weeks of TIL harvest with new brain metastases. Only one patient did not receive ipilimumab after TIL infusion due to dose-limiting colitis following the second (pre-TIL) dose that required ipilimumab discontinuation.

### Clinical Response

**Figure 2:**
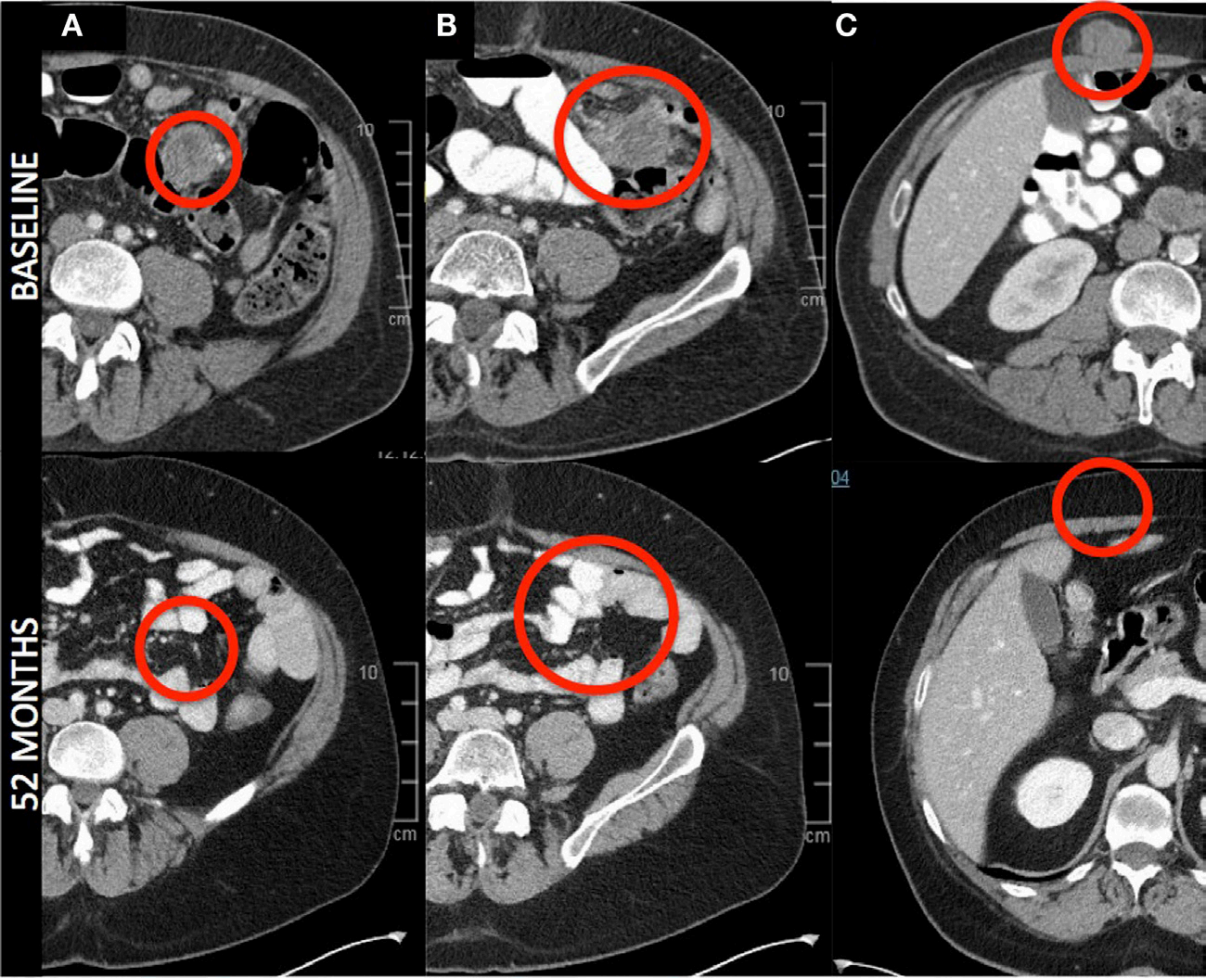
Radiographic response—axial images from a patient with a complete response. Images denote complete resolution of disease in the mesentery **(A,B)**, and abdominal wall **(C)**. All other lesions (liver and posterior abdominal wall, not pictured) completely regressed.

**Figure 3:**
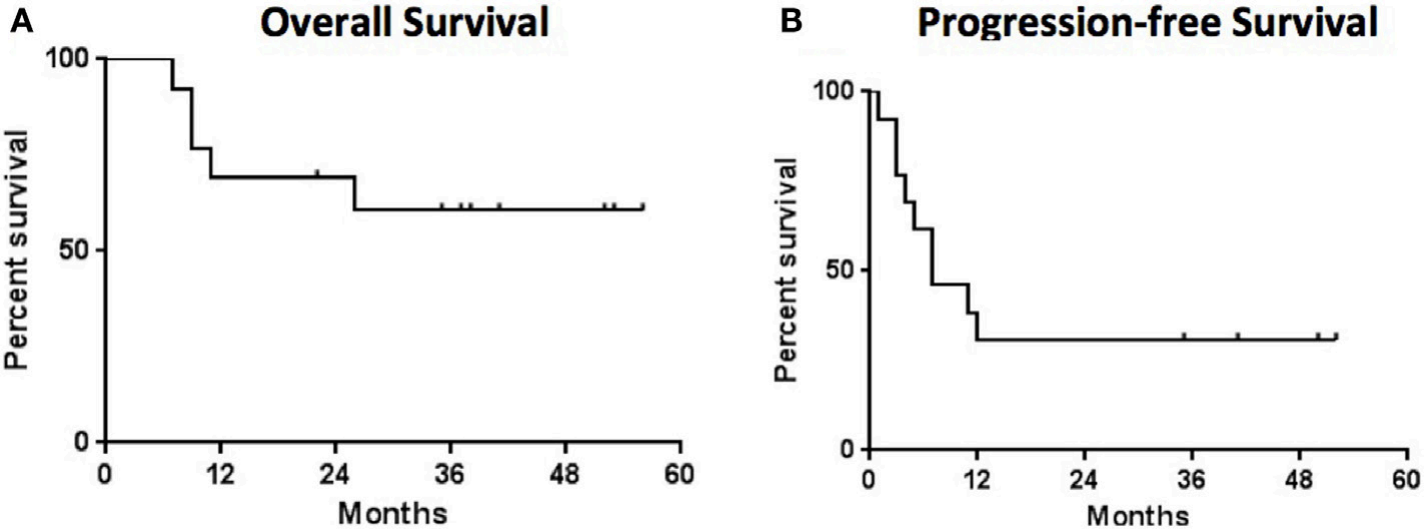
Survival analysis—the median overall survival **(A)** was not reached and the median progression free survival **(B)** was 7 months. Median follow-up for all patients was 35 months.

The secondary endpoint of response at 12 weeks was also assessed (Table 1). The overall objective response rate at 12 weeks was 38% (5 partial responses among 13 patients enrolled) by intention to treat analysis. Of the partial responses, four (80%) are ongoing with durations of 34 +, 38 +, and 46 + months, and one that became a complete response at 52 months and is ongoing at 53 months (Figure 2). This patient had no subsequent treatment after enrollment on this trial. Three patients experienced stable disease at 12 weeks, but all of them experienced disease progression prior to 1 year. The response rate at 1 year was 31%. None of the patients with an ongoing response have had any other treatment aside from the protocol-specified treatment. The median progression-free survival was 7.3 months (95%CI 6.13–29.9 months) and the median overall survival was not reached at a median follow-up of 35 months (Figure 3).

**Figure 4:**
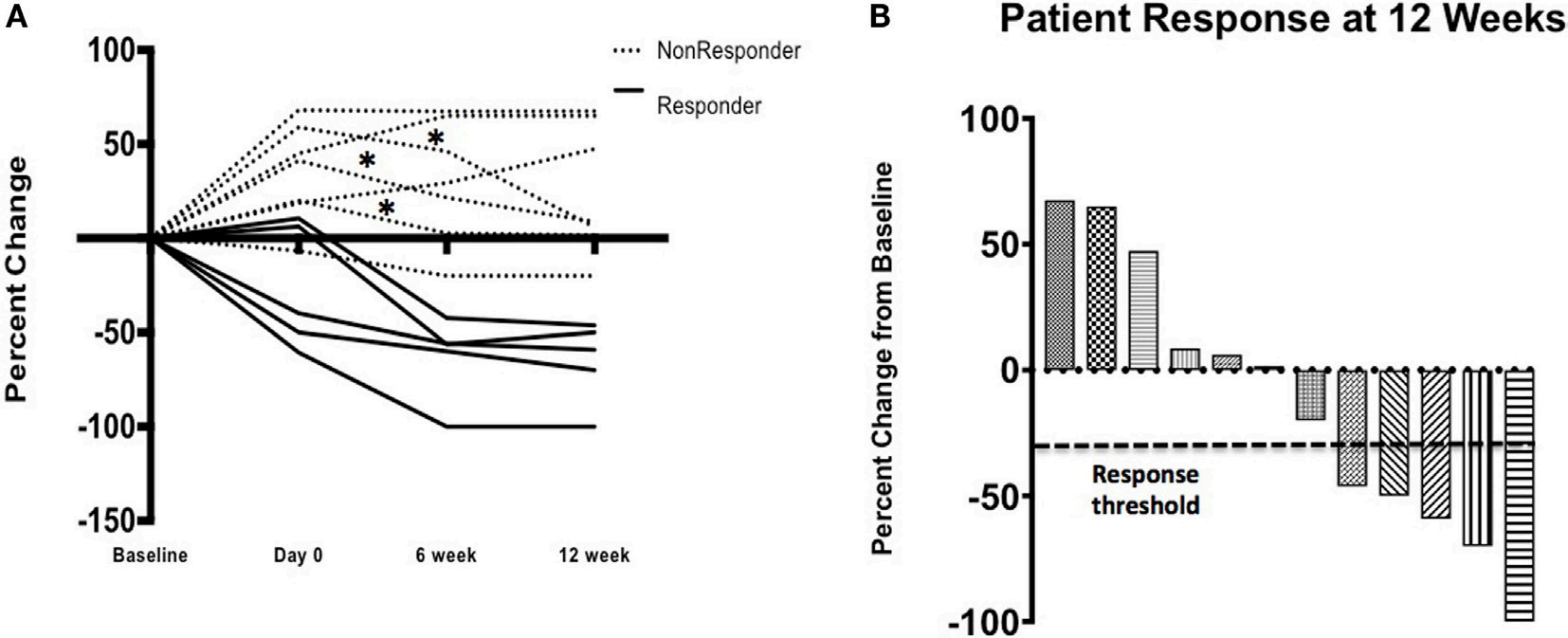
Clinical response—patient response over time **(A)** on study where three (*) patients initially progressed on ipilimumab alone and then had a decrease in the size of their target lesions after cell infusion after initial progression following ipilimumab alone. Waterfall plot **(B)** demonstrates response, by patient, at 12 weeks by RECIST v1.1 criteria.

Twelve patients received TIL and nine (75%) experienced a decrease in target lesion size at week 12 compared with the pre-TIL infusion restaging study. This number includes three patients who were already responding to ipilimumab, three patients who were stable after two doses of ipilimumab and three patients who had experienced some progression by day 0. This interval progression did not prevent TIL infusion. Interestingly, three patients (25%) experienced a mixed response, with growth in some target lesions but regression in others (Figure 4).

### Toxicity

The majority of the toxicity seen in this trial was associated with the lymphodepletion regimen and/or IL-2, which is given every 8 h after TIL infusion for 15 doses or until toxicity mandated discontinuation. Patients received a median of 5 IL-2 doses (range 2–8). Two patients received only two doses of IL-2, and both stopped due to gastrointestinal toxicity. The reason for cessation of IL-2 in the other patients included hypoxemia (6) and hypotension (5). Seven patients suffered Grade ≥ 3 immune-related adverse events likely due to ipilimumab, including hypothyroidism (3), hepatitis (2), uveitis (1), and colitis (1). Adverse events are summarized in Table 2.

**Table 2:** Adverse events.

Adverse event	Grade 3	Grade 4
Anemia	4	
Bacteremia	2	
Cardiac dysrhythmia (NOS)		
Central catheter-related thrombosis		2
Colitis	1	
Dehydration	2	
Electrolyte disturbance	7	
Febrile neutropenia	10	
Hypotension	4	
Hypothyroidism	3	
Leukopenia		12
Pancreatitis		1
Presyncope/syncope	1	
Pulmonary edema	1	
Rash	1	
SIADH	1	
Thrombocytopenia	4	7
Transaminitis	2	
Uveitis	1	

### Properties of TIL Infusion Product

**Figure 5:**
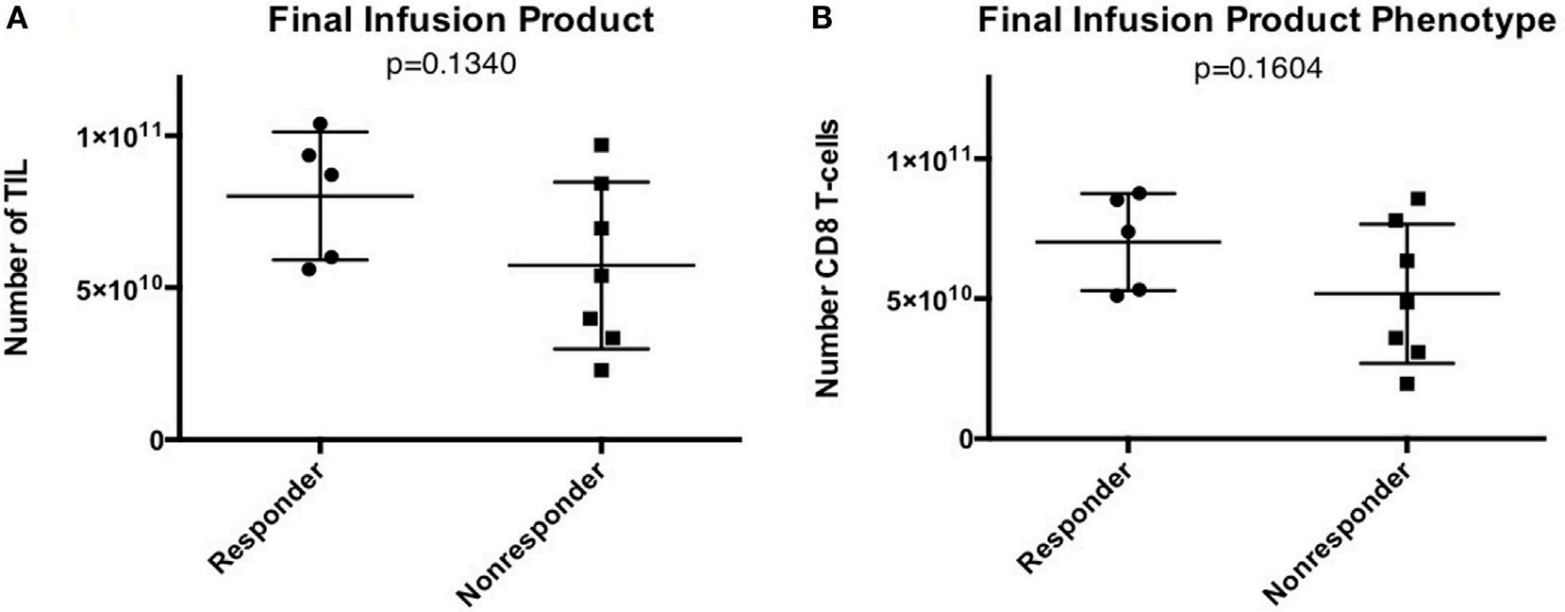
Infusion product characteristics—no difference in tumor-infiltrating lymphocyte (TIL) numbers or phenotype in patients that responded to TIL adoptive cell therapy (ACT). **(A)** Total number of TIL infused. **(B)** Total number of CD8 + T cells in the final infusion product.

All harvested tumors yielded sufficient TIL numbers for rapid expansion, resulting in successful cell expansion in 100% of patients. The TIL grown from 9 of 13 patients (69.2%) had a tumor-specific response when tested by ELISA assay for IFN-γ release (Table 3). Patients received TIL at a mean of 51 days (range 39–77) after tumor harvest and a mean of 6.7 × 10^10^ cells were infused (range 2.29 × 10^10^ to 1.04 × 10^11^). The mean viability of TIL in the infusion product was 89.3% and the mean CD4 + T cell and CD8 + T-cell percentages were 23 and 76%, respectively (Table 4). There was no significant difference in the total number of lymphocytes (*p* = 0.13) or CD8 + T cells (*p* = 0.16) in the final infusion product between responders and non-responders (Figure 5).

**Table 3:** Tumor-infiltrating lymphocyte (TIL) culture characteristics.

Patient number	No. of fragments cultured	No. of fragments grown to >6 wells	No. of fragments tested	No. of fragments tumor-reactive
1	48	38	12	12
2	48	29	12	1
3	48	34	11	11
4	40	25	12	8
5	36	17	12	3
6	48	19	12	4
7	48	14	6	3
8	48	8	8	8
9	48	32	18	18
10	60	11	Post rep	0
11	48	13	Post rep	0
12	48	23	11	6
13	48	28	12	0

Total	616	291	126	74

**Table 4:** Tumor-infiltrating lymphocyte (TIL) infusion characteristics.

Patient number	Days until infusion	Fold expansion	No. of TIL infused	Viability (%)	%CD4	%CD8
1	45	1,733	1.04E + 11	88.4	4	96
2	52	1,453	8.72E + 10	92.5	4	96
3	53	1,002	6.01E + 10	91.3	26	73
4	45	1,560	9.36E + 10	82.0	3	97
5	45	935	5.61E + 10	84.7	31	70
6	46	1,617	9.70E + 10	88.6	73	27
7	59	1,405	8.43E + 10	93.7	2	98
8	39	1,218	6.96E + 10	91	3	97
9	77	900	5.40E + 10	90.1	2	99
10	53	558	3.35E + 10	92.2	53	44
11	53	382	2.29E + 10	86.2	34	56
12	40	665	3.99E + 10	90.3	41	54
13	Not treated	Not treated	Not treated	Not treated	Not treated	Not treated

Mean	50.6	1,119	6.68E + 10	89.3	23.0	75.6

### TCR-Sequencing Data

**Figure 6:**
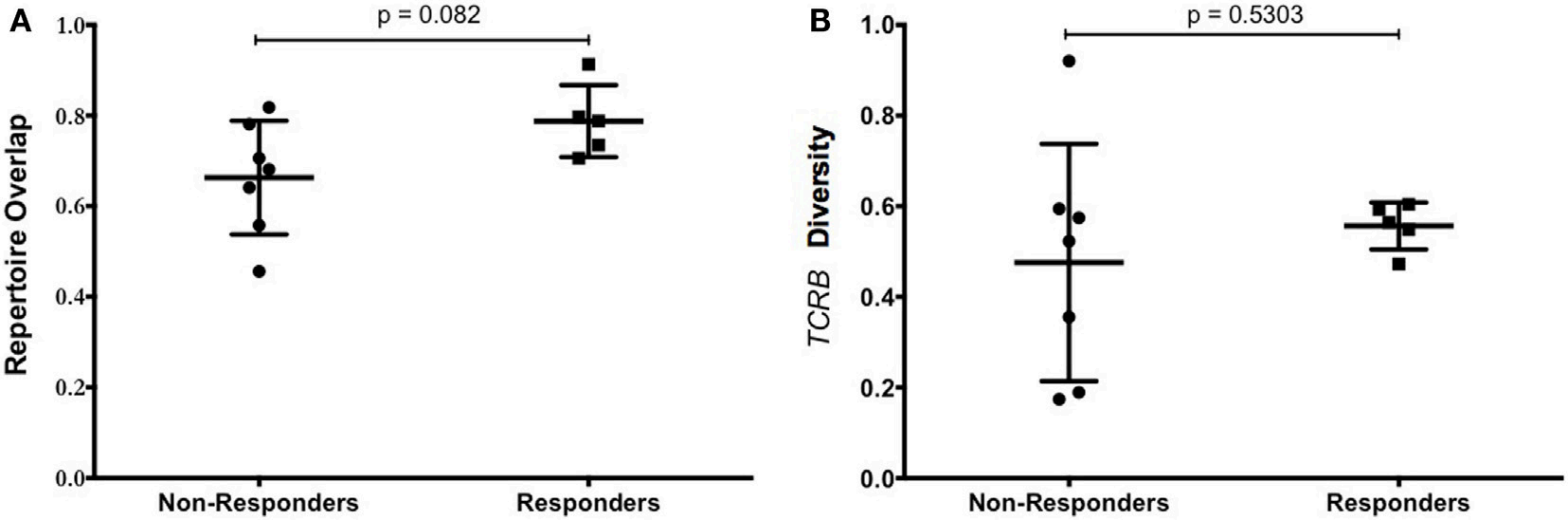
T-cell repertoire of patients. **(A)** Tumor-infiltrating lymphocyte (TIL) persistence did not correlate with objective response. The overlap of unique TCRβ clonotypes contained in the infused TIL product and PBMC at 6 weeks after infusion is shown for patients that responded or progressed after adoptive cell therapy (ACT). **(B)** Diversity of TCRβ repertoire in the infusion product of patients treated with TIL ACT. Patient responses at 12 weeks were plotted against the diversity of the TCRβ repertoire in the TIL infusion product. The diversity of the infusion product was no different between responders and non-responders.

Persistence of transferred TIL in the peripheral blood has been shown to correlate with clinical response (20). To measure the persistence of infused TIL, the distribution of individual T-cell clonotypes was measured in the TIL infusion product and in PBMC obtained 6 weeks after adoptive transfer. The proportion of shared clonotypes was defined as “overlap,” and is indicative of the persistence of the infused TIL product. The overlap between samples was compared between responders and non-responders. As shown in Figure 6A, while there was no significant difference between the groups, there was a trend that increased overlap of the TCRβ repertoire was associated with clinical response to TIL therapy.

The number of different clonotypes at each time point indicates the diversity of the TIL product, and this was evaluated using the range of TCRβ sequences present in the infusion product. There was no difference in the diversity of TCRβ sequences in the infusion product of patients who responded to TIL therapy compared with patients who did not respond (Figure 6B). Despite this, there was a greater distribution of diversity in those that did not respond, indicating a more variable cell product between patients. Total numbers of unique clonotypes and diversity in the TIL infusion product for each patient is shown in Table 5.

**Table 5:** Number of unique clonotypes and diversity of infused tumor-infiltrating lymphocyte (TIL) product, as measured by clonality.

Patient	Response at 12 weeks	Unique, productive clonotypes in TIL	Clonality of TIL
1	PR	16,586	0.4727
2	PR	9,542	0.5484
3	PR	9,365	0.6042
4	PR	16,746	0.5646
5	PR	17,757	0.5929
6	SD	18,875	0.5745
7	SD	1,817	0.9205
8	SD	11,562	0.5943
9	PD	15,839	0.5227
10	PD	31,089	0.3553
11	PD	24,847	0.1894
12	PD	84,408	0.1741

## Discussion

Despite recent advances in the treatment of metastatic melanoma, improvements are still needed. Here, we present the first prospective clinical trial using a combination of an immune checkpoint inhibitor plus ACT with TIL. This strategy has the potential to enhance ACT by decreasing the patient dropout rate, increasing the immune infiltration into the tumor, enhancing the clinical efficacy of TIL through direct interaction with the infused lymphocytes, or a combination of these improvements.

Attrition of patients due to progression prior to TIL transfer has been a limitation of ACT. During the cell preparatory phase of traditional ACT, from tumor harvest to TIL infusion, patients are not treated with any therapy. In our first report of ACT, 6 of 19 patients (32%) did not receive the TIL infusion due to progression following tumor harvest. In this report, attrition due to progression was only 7% (1/13). There were no new safety signals from combination therapy. The trial therefore met its prespecified primary endpoint of safety and feasibility. Furthermore, there was promising efficacy signal observed. At 12 weeks, there was a 38% objective response rate based upon intent to treat, compared with 26% seen in our initial trial using only TIL for ACT. The results seen in this trial were durable, with 80% of responses lasting more than 2 years.

In our experience using TIL alone for patients with advanced melanoma, the success rate for TIL culture from resected tumors was 84%, whereas in this report we were able to generate a TIL culture from all 13 patients. Despite this improvement in our TIL culture success rate, ipilimumab before ACT did not improve the fold expansion, viability, or number of TIL infused compared our prior experience. This is consistent with retrospective reports from other institutions where ipilimumab administration prior to tumor harvest did not affect the yield or phenotype of the cell product (19). In another retrospective report, which included patients with progression after ipilimumab, there was no correlation with the clinical response after ACT relative to ipilimumab-naïve patients (7).

In this trial, patients were treated with one dose of ipilimumab prior to tumor resection for TIL harvest. We hypothesized that pretreatment with ipilimumab would increase the number of tumor-specific T cells infiltrating tumors and lead to improved expansion of TIL *in vitro*. While TIL were expanded from all of the resected tumors in all patients, there was no significant improvement in the reactivity of TIL to autologous tumor in comparison to our initial trial (data not shown). This suggests that one injection of ipilimumab may not be sufficient to expand the repertoire of tumor-specific T cells within tumors. While ipilimumab was given to patients prior to ACT as a means to delay disease progression, it is possible that the preparatory chemotherapy regimen could ablate the effect of initial ipilimumab doses. In our trial, since patients underwent imaging prior to lymphodepletion (after the two initial ipilimumab doses), we can show that all of the patients with an initial response to ipilimumab continued to respond after infusion of TIL, suggesting that non-myeloablative therapy does not prevent patients from responding to the combination of checkpoint blockade and ACT.

The impact of ipilimumab on the expansion and persistence of adoptively transferred TIL is unknown. It has been previously described that patients who responded to TIL therapy demonstrated an increase persistence of adoptively transferred TIL (20). We demonstrated that while there was a trend to increased persistence of TIL in patients who responded in this trial compared with those who did not, the difference was not significant. Additional studies need to be performed to determine whether concurrent checkpoint inhibition contributes to improved persistence of TIL after ACT.

Attrition on clinical trials can increase as the complexity of therapy increases since toxicities can compound. This is especially true of immunotherapy trials, as toxicity often precludes completion of therapy with multi-agent regimens. This was not seen in the present trial as only three patients demonstrated Grade ≥ 3 immune-related toxicity. Additionally, all patients received at least two doses of ipilimumab and 53.8% (7/13) received all four prescribed doses, demonstrating the successful feasibility of this approach.

This is the first prospective clinical trial using checkpoint inhibition combined with ACT. With this approach, more patients may be able to complete the therapy and receive TIL infusion, thereby potentially increasing the objective response rate by an intention to treat analysis. Direct comparison between these patients who received checkpoint inhibition and those in prior studies who received TIL alone is not possible. However, the patients in this trial were checkpoint-naïve, which is certainly not the case for trial-eligible metastatic melanoma patients at this time. Because of this, currently accruing ACT trials at this institution are designed to include patients that have failed checkpoint inhibitors and also incorporate more recently approved checkpoint inhibitors, specifically anti-PD1 antibodies. Data from these trials will be used to validate the fundamental conclusion from this trial that addition of checkpoint inhibitors to the treatment regimen of ACT is feasible and may increase the efficacy of TIL adoptive transfer for patients with advanced melanoma.

## Ethics Statement

This study was carried out in accordance with the recommendations of the Institutional Review Board at Moffitt Cancer Center with written informed consent from all subjects. All subjects gave written informed consent in accordance with the Declaration of Helsinki. The protocol was approved by the Institutional Review Board at Moffitt Cancer Center.

## Author Contributions

AAS, JW, and SP-T contributed to the design of the study. JEM, AAS, SP-T, JJM, JW, and VKS drafted the manuscript. JEM, MH, AAS, SP, ER, AR, VS and SP-T analyzed the data. JW, AAS, NK, ZE, AB, JM, LK, CC, JSZ, SP, and VKS collected the data. All authors approved the final version of the manuscript and agreed to be accountable to all aspects of this work.

## Conflict of Interest Statement

JW serves on the Advisory Board for Bristol Myers-Squibb and holds a patent for a biomarker to ipilimumab response. He also serves on Advisory Boards for Merck, Genentech, Astra Zeneca, GSK, Novartis, Sellas, CytoMx, Altor, Nektar, Medivation, Celldex, Incyte, Jounce, WindMIL, and EMD Serono. JM serves on the Scientific Advisory Board for Iovance Biotechnologies, Inc. VS is a compensated consultant for Array, Bristol Myers-Squibb, Genentech, Merck, Novartis and Polynoma. All other authors report no conflicts of interest. The reviewer VH and handling editor declared their shared affiliation.
